# Induction of an EMT-like transformation and MET *in vitro*

**DOI:** 10.1186/1479-5876-11-164

**Published:** 2013-07-07

**Authors:** Songming Ding, Wu Zhang, Zhiyuan Xu, Chunyang Xing, Haiyang Xie, Haijun Guo, Kanjie Chen, Penghong Song, Yu Gu, Fengqiang Xiao, Lin Zhou, Shusen Zheng

**Affiliations:** 1 Key Laboratory of Combined Multi-organ Transplantation, Ministry of Public Health; Key Laboratory of Organ Trans-plantation, Hangzhou, Zhejiang, China; 2 Division of Hepatobiliary and Pancreatic Surgery, Department of Surgery, First Affiliated Hospital, Zhejiang University School of Medicine, Hangzhou, Zhejiang, P.R. China

**Keywords:** Tumor microenvironment, EMT, MET, Co-culture, Conditioned medium culture

## Abstract

**Background:**

The epithelial-to-mesenchymal transition (EMT) and mesenchymal-to-epithelial transition (MET) play pivotal roles in metastasis of epithelial cancers. The distinction between them has shed new light on the molecular mechanisms of tumor metastasis. Recently, tumor microenvironment (TM) has been identified as one of the most potent inducers of EMT and MET. TM is characterized by its complexity and flexibility. The purpose of this study was to ascertain the exact effect of each distinct TM component on the evolution hepatocellular carcinoma (HCC) metastasis.

**Methods:**

Two different cell culture models were used. The HCC cell line Bel-7402 was co-cultured with the normal liver cell line HL-7702 or with the retinal vascular endothelial cell line RF/6A in double-layer six-well plates, imitating the direct interaction between tumor-host cells and tumor cells. Bel-7402 was also cultured in the conditioned medium (CM) of the human lung fibroblast cell line MRC-5, HL-7702 or RF/6A, imitating an indirect interaction. Integrin β1, β3, β4, β7, laminin β3, E-cadherin and Snail levels were measured by quantitative RT-PCR in tumor sepecimens from 42 resected HCC.

**Results:**

We found that Bel-7402 cells co-cultured with HL-7702 or RF/6A cells were induced to undergo MET. The expression of E-cadherin, α-catenin and β-catenin was up-regulated, accompanied with a strengthened E-cadherin/catenin complex on the membrane of co-cultured Bel-7402 cells. Consequently, the invasion and migration ability of cells was declined. Conversely, Bel-7402 cells cultured in conditioned medium from MRC-5 cells underwent an EMT-like transformation as the cells became elongated with increased invasion and migration ability. Furthermore, we demonstrated that HL-7702 cells could generally inhibit the tumorigenicity and viability of Bel-7402 cells. We also found that integrin β1 expression was negatively associated with capsular formation, and that integrin β4 expression was negatively associated with CK19 expression.

**Conclusion:**

Our findings highlight the strong influences exerted by TM on tumor progression through EMT and MET by impacting the expression of adhesion molecules, including the E-cadherin/catenin complex, laminins and integrins.

## Background

The tumor microenvironment (TM), comprised of microvascular endothelial cells, adjacent normal epithelial cells, and tumor-associated fibroblasts, governs the fate of cancer cells [1-3]. TM promotes tumorigenesis and angiogenesis [4,5] and is the inducer of the epithelial-to-mesenchymal transition (EMT) and mesenchymal-to-epithelial transition (MET). EMT is involved in metastasis of many types of cancer [6-11], and cancer cells that undergo EMT have increased invasion and migration ability [12,13]. Molecular markers of EMT include decreased expression of E-cadherin/catenin complex, increased expression of N-cadherin, vimentin and matrix metalloproteinases (MMPs), and increased production of transcription factors such as Snail, Slug, Twist1, Gli-1, ZEB-1 and ZEB-2. MET is also implicated in tumor metastasis [14-17]. Tumor cells that undergo MET lead to distal re-epithelialization metastases and re-expression of the E-cadherin/catenin complex is the only accepted criterion for defining MET [18].

Signaling pathways regulating EMT and MET have been widely studied. Hedgehog, Notch, Wnt, and transforming growth factor (TGF)-β are capable of initiating EMT via up-regulation of a group of transcription factors [19] and a large number of laminins [20] and integrins [21] related to EMT have been identified. However, the precise mechanisms of EMT/MET remain to be elucidated. Thus, it is imperative to distinguish the *ad hoc* impact of each TM component on the invasive and migratory potential of cancer cells, to better understand the underlying mechanisms of EMT/MET.

The process of tumor metastasis consists of a series of orderly and interrelated steps [22]. Each step is orchestrated but rather rate-limiting. The outcome of the process is influenced by the interaction between cancer cells and the local microenvironment. Conceivably, metastasis formation is affected by the TM [23]. The “seed and soil” theory states that EMT is pivotal to the former stages of cancer metastasis, and MET is critical to the latter stages. Therefore, it is compelling to discriminate which component of TM can induce EMT and which can induce MET, to better understand the sequential steps of metastatic progression.

In this study, we used a co-culture model and a conditioned media (CM) model to simulate the interactions between tumor-host cells and tumor cells. We observed the biological behavior change of cancer cells and further explored the associated molecular mechanisms.

## Methods

### Patients

42 HCCs who underwent curative hepatic resection between 2009 and 2011 were recruited from our center. Specimens were obtained promptly after surgical resection. None of patients were treated by any preoperative therapy. These patients included 37 males and 5 females with a mean age of 57.1 ± 12.1 years (range, 33–82 years). The study was approved by the ethics committee of our hospital.

### Cell culture

RF/6A cells were kindly gifted from Dr. Panpan Ye (Zhejiang University, China) and MRC-5 cells were kindly gifted from Dr. Xi Chen ( Zhejiang University, China). Bel-7402 and HL-7702 cells were purchased from Shanghai Cell Bank, Chinese Academy of Sciences.

Co-culture model: The double-layer six-well plates were purchased from Corning (Polyester Membrane Transwell-clear Inserts, 0.4 μm pore size). Bel-7402 cells (5× 10^4^) were cultured on the upper-layer of the double-layer six-well plate and HL-7702 or RF/6A cells (5× 10^4^) were cultured on the lower-layer of the double-layer six-well plate at the initial stage of co-culture. Upper and lower cells could not penetrate the polyester membrane. Cells were maintained in RPMI-1640 (Gibco) containing 10% FBS (fetal bovine serum) (Sigma-Aldrich) and incubated at 37°C in a humidified environment containing 5% CO2. Culture medium was changed every 4–5 days. When co-cultured cells reached 80%-90% confluence, they were digested using 0.25% trypsin and were collected respectively. The double-layer six well plates were washed gently three times using PBS. Then, 5× 10^4^ Bel-7402 had been co-cultured were re-plated on the upper layer and 5× 10^4^ co-cultured HL-7702 or RF/6A were re-plated on the lower layer. The remaining cells were used for western-blot analysis, transwell assay, confocal immunofluorescent analysis, extracting the total RNA, in vitro colony formation assay or in vivo tumorigenicity experiment. Bel-7402 cells were co-cultured with HL-7702 or RF/6A cells for 90 days (n = 2).

Conditioned media models: MRC-5, HL-7702 and RF/6A cells were grown in RPMI-1640 (Gibco) supplemented with 10% FBS (fetal bovine serum) (Sigma-Aldrich) and maintained at 37°C in a 5% CO2 water-saturated environment. To collect the conditioned media from MRC-5 cells, the cells were cultured until 70%-90% confluence. Then, the used media were harvested and passed through a 0.22 μm fliter and diluted at a ratio of 1:1 with RPMI-1640 containing 10% FBS. Collect the conditioned media from HL-7702 and RF/6A cells through similar procession. RPMI-1640 medium supplemented with 10% FBS serverd as a control and was named unconditioned medium. Bel-7402 cells were cultured in the conditioned media for 28 days (n = 3). Bel-7402, HL-7702 and RF/6A cells were subcultured once a week at a ratio of 1:3 or 1:5. MRC-5 cells were subcultured once a week at a ratio of 1:1 or 1:2.

### Transwell assay

The assays were performed using Transwell chambers (8 μm pore size; Millipore, Billerica, MA,USA) coated with matrigel (BD Bioscience, San Jose, CA, USA). 3× 10^4^-5× 10^4^ cells were placed in the upper chamber. The crystal violet staining cells were counted under an inverted microscope.

### Western blot analysis

Proteins were extracted with RIPA (Beyotime, Shanghai, China), separated by 10%-12% NUPAGE Bis-tris Gel (Invitrogen, CA, USA) and transferred onto polyvinylidene difluoride membranes. The primary antibodies were detailed in Additional file 1: Table S1.

### F-actin immunofluorescence and confocal immunofluorescent analysis

5× 10^5^ cells were implanted onto a cell culture dish for 24 hours (NEST Biotech, Hong Kong, China). F-actin immunofluorescence: Cells were fixed with paraformaldehyde for 30 minutes, washed three times with PBS then permeabilized with 0.1% Triton X-100 for 10 minutes at room temperature, washed three times with PBS and thereafter incubated with Rhodamine-conjugated phalloidin (5 μg/ml) (Sigma-Aldrich) in the dark for 1 hour at room temperature.

Confocal immunofluorescent analysis: Cells were fixed with paraformaldehyde for 30 minutes, washed three times with PBS then permeabilized with 0.1% Triton X-100 for 10 minutes at room temperature, washed three times with PBS and thereafter sealed with goat serum for 1 hour at room temperature following primary antibodies incubation in the dark for 24 hours at 4°C. Washed three times with PBS and then cells were incubated with Alexa Flour® 488 IgG donkey anti-mouse or anti-rabbit second antibodies (1:300, Invitrogen, USA) in the dark for 1 hour at room temperature. Then, nuclei were stained with propidium iodide for 5 minutes. Fluorescence images were photographed with confocal microscopy (Leica DMIRE2, Germany).

### Colony formation assay

1× 10^3^ cells were plated in a 8 cm plate. After two weeks of culture, the colonies (>10cells) were stained with crystal violet and counted. Colony formation efficiency was defined as the ratio of the number of colonies formed in the culture to the number of cells inoculated.

### Tumorigenicity experiment

Tumor xenografts were generated by injection of 2 × 10^6^ Bel-7402 cells, 2 × 10^6^ Bel-7402 cells co-cultured with HL-7702 cells for 74 days and 2 × 10^6^ Bel-7402 cells cultured in conditioned medium of MRC-5 cells for 28 days into the subcutaneous tissue of the axillary region of nude mices, and mices were dissected 4 wk later (n = 2).

### Transfection

Bel-7402 cells were transfected with miR-200a mimics (GenePharma) or negative control using Lipofectamine 2000 (Invitrogen) and were harvested 48 h later.

### Quantitative reverse-transcription-PCR

Total RNA from tissues and cells was extracted using Trizol reagent (Invitrogen) and reverse-transcribed with M-MLV Reverse Transcriptase (Promega). The primers for q-RT PCR were purchased from Biosune, the SYBR® Premix Dimmer Eraser kits were from TaKaRa (TaKaRa, Dalian, China).

GAPDH was used as an internal control to normalize target mRNA level. qRT-PCR reactions were performed by ABI7500 (Applied Biosystems, CA). The relative expression fold change was calculated by the 2^-ΔΔCt^ method.

### Statistical analysis

Statistical analysis was performed using SPSS 16.0 software (SPSS, Chicago, IL, USA). Chi-square test was used for associations between the expression of EMT-related genes and different clinicopathologic parameters. Independent t test was applied to analyze the differences between 2 groups and one-way ANOVA test was applied to analyze the differences in xenograft tumor volumes. Statistical significance was accepted if *p* < 0.05.

## Results

### Co-culture of Bel-7402 with HL-7702 or RF/6A suppressed the invasion and migration of Bel-7402

**Figure 1:**
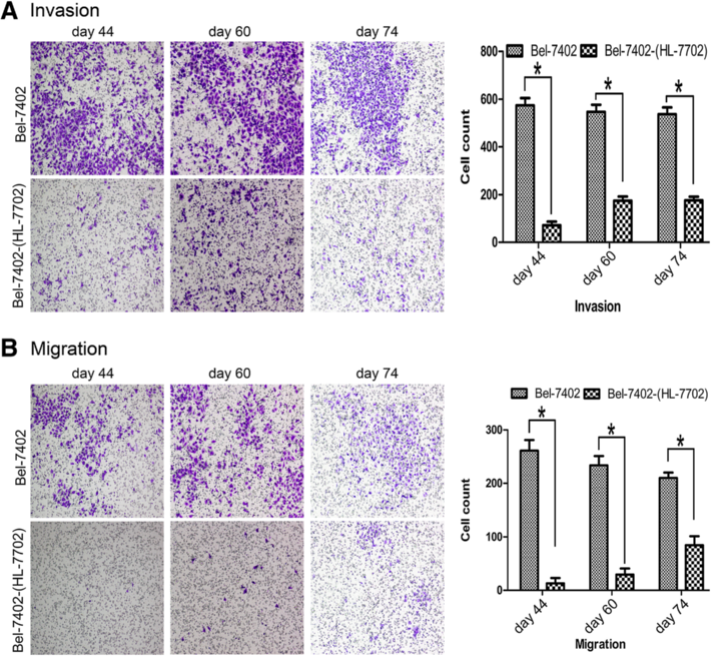
**Invasion and migration of Bel-7402 and Bel-7402-(HL-7702).** A transwell invasion **(A)** and migration **(B)** assay of Bel-7402 cells after co-culture with HL-7702 for 44, 60, or 74 days. Invasion and migration of co-cultured Bel-7402 cells were impaired (*p* < 0.05).

**Figure 2:**
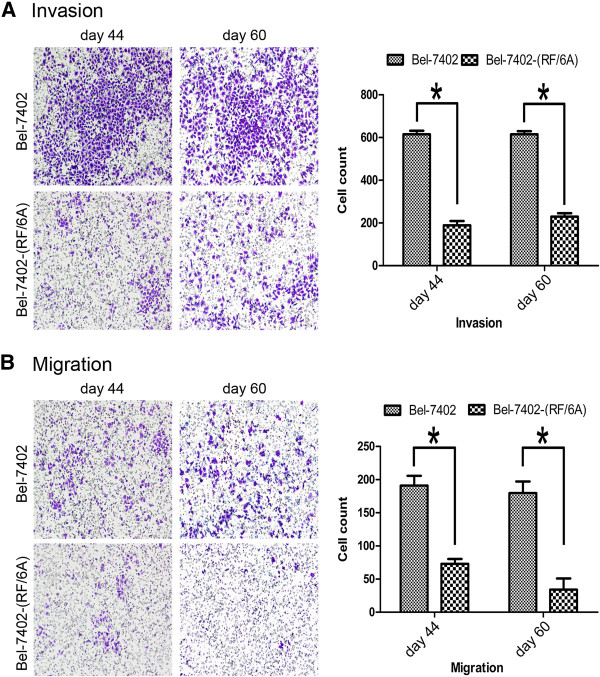
**Invasion and migration of Bel-7402 and Bel-7402-(RF/6A).** A transwell invasion **(A)** and migration **(B)** assay of Bel-7402 cells after co-culture with RF/6A for 44 and 60 days. Invasion and migration of co-culture Bel-7402 declined (*p* < 0.05).

Bel-7402 cells were co-cultured with either HL-7702 cells [Bel-7402-(HL-7702)] or RF/6A cells [Bel-7402-(RF/6A)] for 90 days. The invasion and migration ability of Bel-7402-(HL-7702) cells were determined by transwell assay at days 44, 60 and 74. In Figure 1, we show that Bel-7402-(HL-7702) cells had poorer invasion and migratory capacity compared with control Bel-7402 cells (*p* < 0.05). Altered invasion and migration of Bel-7402-(RF/6A) cells was also measured using the transwell assay at days 44 and 60. In Figure 2, we show that Bel-7402-(RF/6A) cells failed to invade and migrate (*p* < 0.05).

### Differential expression of EMT-related genes in Bel-7402 co-cultures

**Figure 3:**
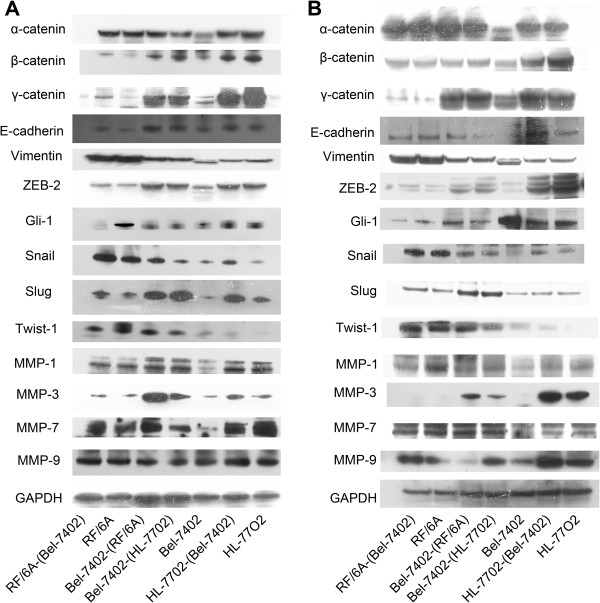
**Epithelial and mesenchymal marker expression.** Western blot analysis of Bel-7402 cells co-clutured with HL-7702 or RF/6A cells for 28 days **(A)** and 44 days **(B)**.

**Figure 4:**
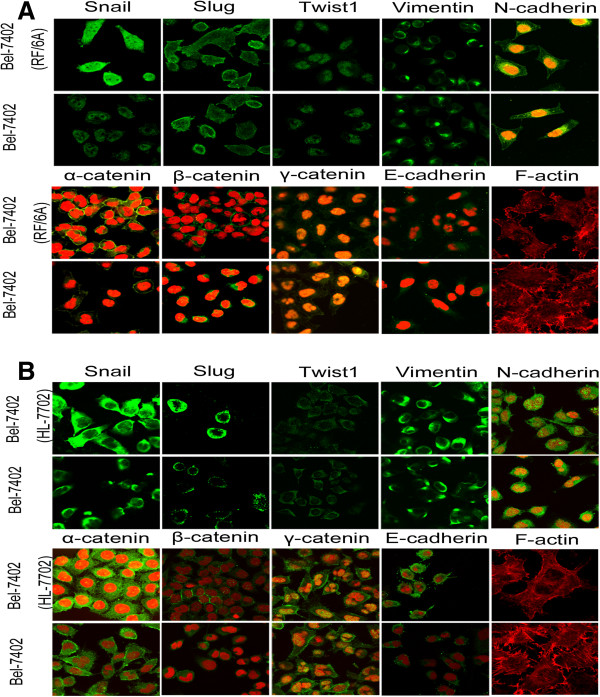
**Immunofluorescence of epithelial and mesenchymal markers, EMT-related transcription factors and F-actin. (A)** Confocal microscopy analysis of Bel-7402 cells co-clutured with RF/6A cells for 72 days. **(B)** Confocal microscopy analysis of Bel-7402 cells co-clutured with HL-7702 cells for 60 days. The green signal represents corresponding protein staining and the red signal indicates nuclear DNA staining by propidium iodide (PI).

To elucidate the mechanism underlying impaired invasion and migration capacity of co-cultured Bel-7402, we evaluated the expression profiles of an epithelial marker (E-cadherin/catenin complex), mesenchymal marker (vimentin), EMT-promoted transcription factors (Snail, Slug, Twsit1, Gli-1 and ZEB-2) and MMPs (MMP-1, 3, 7, 9) by Western-blot at days 28 and 44 (Figure 3). The E-cadherin/catenin complex was up-regulated in co-cultured Bel-7402 cells compared to Bel-7402 cells that were not co-cultured. The results also revealed that the expression levels of mesenchymal markers such as Snail, Slug, Twsit1, ZEB-2, MMP-3, MMP-7 and vimentin were higher in co-cultured Bel-7402 cells. Interestingly, expression of the EMT-related transcription factor Gli-1 was decreased. In addition, the expression level trend of MMP-1 and MMP-9 in co-cultured Bel-7402 cells was unstable. A portion of these results was confimed by confocal microscopy, as shown in Figure 4. Moreover, filamentous actin (F-actin), which plays an important role in cell motility, was decreased in co-cultured Bel-7402 cells, suggesting that cellular motility of co-cultured Bel-7402 cells was reduced.

### Effect of conditioned medium (CM) on Bel-7402

**Figure 5:**
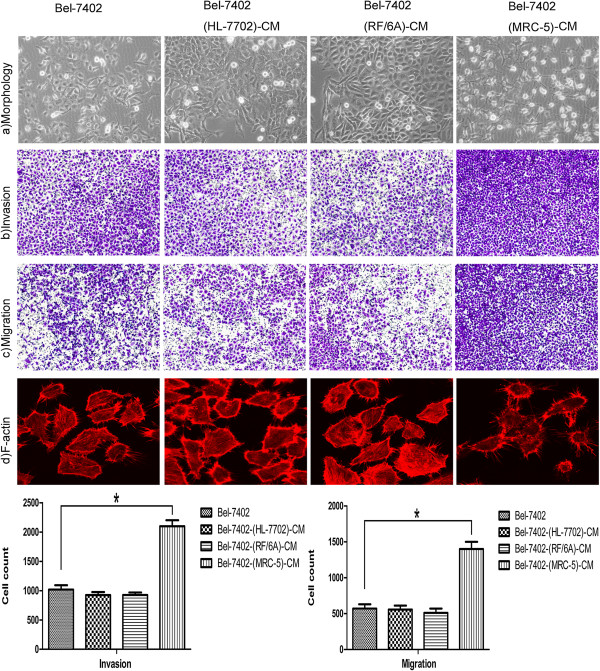
**Establishment of the conditioned medium (CM) models.** CM of MRC-5 cells induced Bel-7402 cells to undergo an EMT-like transformation, while CM of HL-7702 cells or RF/6A cells did not induce such a transformation at day 14. **(A)** Bel-7402 cells cultured in CM of MRC-5 cells became elongated. **(B)** and **(C)** The invasion and migration ability of Bel-7402 cells cultured in CM of MRC-5 cells was increased (*p* < 0.05). **(D)** Bel-7402 cells cultured in CM of MRC-5 cells had more actin-rich protrusions.

We used CM from MRC-5, HL-7702 and RF/6A cells to culture Bel-7402 cells for 28 days. Bel-7402-(MRC-5)-CM cells were unrecognizable at day 14 (Figure 5), with an elongated and spindle-like morphology containing actin-rich protrusions. We further evaluated the invasion and migration ability of Bel-7402-(MRC-5)-CM cells. The results in Figure 5 show that the invasion and migration ability of Bel-7402-(MRC-5)-CM cells was increased significantly (*p* < 0.05). In contrast, the morphology and motility of Bel-7402-(HL-7702)-CM and Bel-7402-(RF/6A)-CM cells did not change significantly compared to Bel-7402 control cells at day 14 (*p* > 0.05). These results indicate that MRC-5 cells induced Bel-7402 cells to undergo an EMT-like transformation while the CM of HL-7702 or RF/6A cells failed to induce Bel-7402 cells to undergo MET.

### Cell viability and tumorigenicity

**Figure 6:**
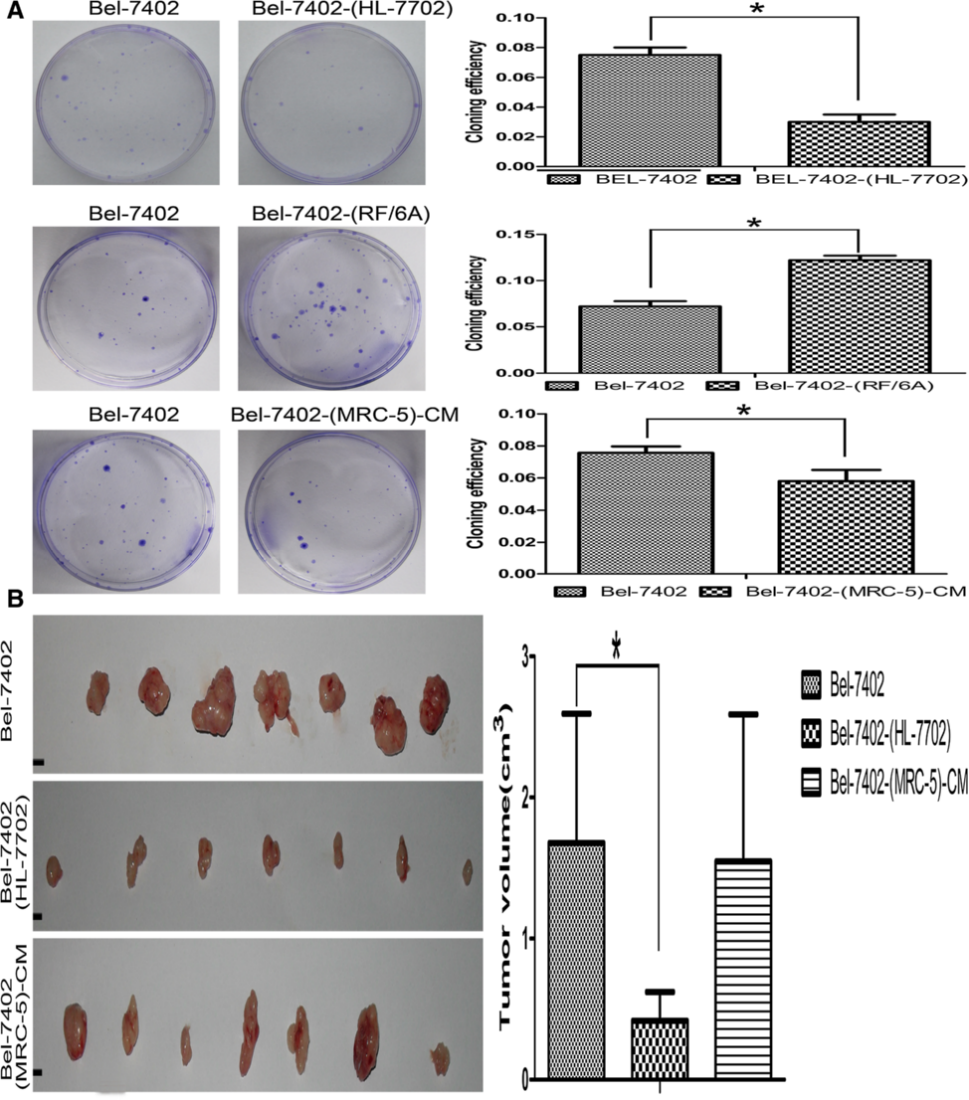
**Cell viability and tumorigenicity. (A)** A colony formation assay evaluated the growth of Bel-7402 cells co-cultured with HL-7702 cells for 74 days, co-cultured with RF/6A cells for 60 days and Bel-7402 cells cultured in CM of MRC-5 for 28 days. **(B)** Tumor forming ability of Bel-7402 cells co-cultured with HL-7702 cells for 74 days and Bel-7402 cells cultured in CM of MRC-5 cells for 28 days were assessed in nude mice by axillary subcutaneous implant (n = 7 per group). Scale bar = 5 mm.

To explore the effects of disparate TM components on tumor cell growth, we performed the colony formation assays (Figure 6A). We identified a significant reduction in the colony formation ability of Bel-7402-(MRC-5)-CM and Bel-7402-(HL-7702) cells compared with Bel-7402 cells (*p* < 0.05). In contrast, colony formation by Bel-7402-(RF/6A) cells was enhanced significantly (*p* < 0.05). Next, we conducted *in vivo* tumorigenicity experiments to determine the definite roles of HL-7702 and MRC-5 cells in tumor development. Tumor xenograft studies in Figure 6B demonstrated that the growth of tumors derived from Bel-7402-(HL-7702) cells was inhibited, as evidenced by a 75% decrease in tumor volume 4 weeks after implantation. However, MRC-5 had no obvious effect on the tumorigenic ability of Bel-7402 (*p* > 0.05).

### Laminin and integrin expression and the effects of miR-200a mimics

Laminins and integrins are intimately related to cell motility and implicated in various aspects of tumor progression. The microRNA (miR)-200 family has been reported to inhibit the EMT. Therefore, we assayed laminin and integrin mRNA levels in Bel-7402, Bel-7402-(RF/6A), and Bel-7402 cells transfected with miR-200a mimics and in Bel-7402-(HL-7702)-CM, Bel-7402-(MRC-5)-CM and Bel-7402-(RF/6A)-CM cells. In Additional file 2: Figure S1, we showed that integrin β4 was down-regulated in Bel-7402-(MRC-5)-CM cells, while laminin α1 and integrin α4, α11, AL, AV, β1, β6, β7, β8 were up-regulated. In contrast, integrin β4 was up-regulated and laminin α1 was down-regulated in Bel-7402-(HL-7702)-CM, Bel-7402-(RF/6A)-CM, as well as in Bel-7402-(RF/6A) and Bel-7402 cells transfected with miR-200a mimics. Additionally, integrin α4, α11, AL, AV, β1, β6, β7, β8 were down-regulated in Bel-7402-(RF/6A) and Bel-7402 cells transfected with miR-200a mimics. This suggests that EMT/MET are related to laminin and integrin expression.

### Expression of EMT/MET markers in HCC tissues

We evaluated the expression of integrin β1, β3, β4, β7, laminin β3, E-cadherin and Snail in 42 pairs of primary HCC and adjacent normal tissues using quantitative RT-PCR. Our results indicated that almost 76% of primary HCC tumors expressed lower levels of E-cadherin compared with the matched adjacent non-tumor tissues (*p* < 0.05); however, there were no significant differences in the expression levels of integrin β1, β3, β4, β7, laminin β3 and Snail (*p* > 0.05, Additional file 3: Figure S2). We next compared the expression of these genes to various clinicopathologic parameters. A clinical association study showed that integrin β1 expression was significantly correlated with age and capsular formation (*p* < 0.05); integrin β3 was significantly correlated with age; and integrin β4 was negatively and significantly correlated with CK19 expression (Additional file 4: Table S2).

## Discussion

In this study, we demonstrated that co-culture of Bel-7402 cells with HL-7702 or RF/6A cells led to initiation of MET and an attenuation of *in vitro* invasion/migration, characterized by increased expression of the E-cadherin/catenin complex. However, the mesenchymal properties were not markedly affected. E-cadherin repressors including Snail, Slug, Twist1 and ZEB-2 were moderately-to-strongly induced, and mesenchymal markers including vimentin, MMP-3 and MMP-7 were significantly induced. Accordingly, co-cultured Bel-7402 underwent only a partial reversion of EMT. These results correlate with previous reports [24,25] demonstrating that the induction or silencing of one or some of the most potent pro-EMT agents did not necessarily impinge on the expression of all of the other EMT markers. This phenomenon could be explained by the complexity of the EMT regulation network.

It is important to keep in mind that EMT is probably a transient and reversible event during tumor progression and could occur at several stages of the metastatic process, such as during the intra- or extravasation [26]. Additionally, since EMT could be initiated by many different signaling effectors in a paracrine fashion, only a minimal number of tumor cells might be responsive to EMT-inducing cues [27]. Furthermore, the classical EMT event is considered as the exception, and the induction of all EMT characteristics within tumors is difficult to observe [28]. To further understand the complex biological processes, such as cell motility and reversion of EMT, we analyzed the expression of vascular endothelial growth factor (VEGF)-c, hepatocyte nuclear factor 4α (HNF4α), CD147 and Sonic hedgehog-Gli pathway components (SHH, Patch-1 and SMO) in co-cultured Bel-7402 cells, all of which are associated with tumor cell invasion and migration [29-32], as shown in (Additional file 5: Figure S3). HNF4α, which could suppress the development of HCC via inhibiting activation of β-catenin, was increased in co-cultured Bel-7402 cells. However, CD147, which could promote cell motility through regulating annexin II-activated RhoA and Rac1 signaling pathways, was also up-regulated. Moreover, Sonic hedgehog-Gli pathway components changed in opposite trends. The expression of VEGF-c did not change. Therefore, the relevance of EMT/MET in human tumors remains to be resolved.

Our studies verified that MRC-5 induced Bel-7402 cells to undergo an EMT-like transformation. These were the only cells that produced a large quantity of hepatocyte growth factor (HGF) and co-expressed the HGF receptor c-Met and HGF activator (HGFA). The HGF/MET signaling pathway is associated with cancer cell migration and invasion [33]. However, the mechanism underlying the induction of Bel-7402 cells to undergo EMT-like transformation remains to be elucidated.

Recently, significant insight had been obtained linking EMT and the acquisition of epithelial stem cell properties [34]. In the present study, we demonstrated for the first time that HL-7702 cells could significantly inhibit the tumorigenic ability and viability of Bel-7402 cells. Contradictorily, RF/6A cells enhanced the colony formation ability of Bel-7402 cells. Intriguingly, CM of MRC-5 did not promote the tumorigenicity of Bel-7402 cells; rather, MRC-5-CM inhibited the colony-forming efficiency of Bel-7402 cells. It appears that microvessels maintain the survival of tumor cells in the blood and could operate as a “seed repertory” for tumor metastasis. This is in contrast to parenchymal cells in tumor-host tissue that might suppress tumor development. Also, MRC-5 fibroblasts might preferentially generate HCC cells with enhance motility. However, whether MRC-5 cells could generate HCC cells with stem cell potential remains a matter for debate.

Laminins and integrins have recently been identified as EMT biomarkers in head and neck squamous cell carcinomas progression [35], suggesting them to be related to invasion and migration of cancer cells. In the present study, we confirmed that the expression of laminin α1 and integrin α4, α11, AL, AV, β1, β6, β7, β8 was significantly associated with the malignant potential of HCC cells. In addition, we evaluated the expression of integrin β1, β3, β4, β7, laminin β3, E-cadherin and Snail in 42 paired HCC surgical tissues. The results showed that down-regulation of integrin β1 was correlated with capsular formation, and that integrin β4 was negatively correlated with CK19 expression. The role of integrin β1 has been well documented in breast cancer [36] and non-small cell lung cancer [37] during the few last years, but to date, its role in HCC progression has received less attention. Moreover, the integrin β1 expression in breast cancer remains controversial. Some studies reported that down-regulated expression of integrin β1 was correlated with more aggressive disease [38], while others demonstrated that up-regulated expression of integrin β1 was correlated with decreased survival [39]. One possible explanation is that the alteration of integrin β1 expression is associated with different stages of tumor progression. Positive expression of CK19 indicated more aggressive HCC and was a valuable predictor of early recurrence and poor prognosis [40]. Expression of integrin β4 showed a significant negative correlation with CK19, suggesting that integrin β4 inhibits the progression of HCC.

The expression of integrin β7, laminin β3, Snail and E-cadherin was frequently decreased in HCC tissues. Expression levels of these factors did not differ significantly according to age, gender, liver cirrhosis, tumor size, CK19, CK34, capsular formation, vascular invasion and hitological differention, likely due to the limited number of cases.

## Conclusion

EMT/MET requires highly dynamic cell-to-cell and cell-to-matrix interactions, which are regulated by adhesion molecules including the E-cadherin/catenin complex, laminins, and integrins. HCC patients with lower integrin β1 expression may be prediposed to capsular formation, and those with lower integrin β4 expression may be prediposed to cancer recurrence and progression. The association between laminin and integrin expression and HCC patient prognosis should be further clarified.

## Consent

Written informed consent was obtained from the patient for the publication of this report and any accompanying images.

## Competing interests

All authors declare no competing interests.

## Authors’ contribution

SD, ZX, WZ, HG, FX, YG, and CX were involved in trial design and execution. SD, HX, KC, PS, LZ and SZ reviewed the literature, wrote the paper, and proofread the final copy. All authors read and approved the final manuscript.

## Supplementary Material

Additional file 1: Table S1 List of proteins tested and characteristics of the antibodies used. Click here for file

Additional file 2: Figure S1 Laminin and integrin expression. (A) Quantitative RT-PCR results of laminin and integrin in Bel-7402 cells and in Bel-7402 cells co-cultured with RF/6A cells for 90 days. (B) Quantitative RT-PCR results of laminin and integrin in Bel-7402 transfected with negative control or miR-200a mimics. (C) Quantitative RT-PCR results of laminin and integrin in Bel-7402 cells, Bel-7402 cells cultured in conditioned media (CM) of RF/6A cells, Bel-7402 cells cultured in CM of HL-7702 cells, and Bel-7402 cells cultured in CM of MRC-5 cells for 28 days. Click here for file

Additional file 3: Figure S2 The relative expression of integrin β1, β3, β4, β7, laminin β3, E-cadherin and Snail in 42 pairs of primary HCC and their adjacent normal tissues. Click here for file

Additional file 4: Table S2 The relationship between the integrin β1, β3, β4, β7, laminin β3, E-cadherin and Snail expression and clinicopathologic features of 42 patients with HCC. Click here for file

Additional file 5: Figure S3 VEGF-c, CD147, HNF4α, SHH, Patch-1 and SMO expression. (A) Immunofluorescence analysis of VEGF-c, CD147, HNF4α, SHH and Patch-1 in Bel-7402 cells co-cultured with RF/6A cells for 72 days. (B) Immunofluorescence analysis of VEGF-c, CD147, HNF4α, SHH and Patch-1 in Bel-7402 cells co-cultured with HL-7702 cells for 60 days. (C) Evaluation of SMO in co-cultured Bel-7402 cells at day 44. Click here for file
