# Supplementary figures and images for: Induction of an EMT-like transformation and MET in vitro
Source: J Transl Med. 2013 Jul 7;11:164. doi: 10.1186/1479-5876-11-164 (PMC3716679; doi:10.1186/1479-5876-11-164)

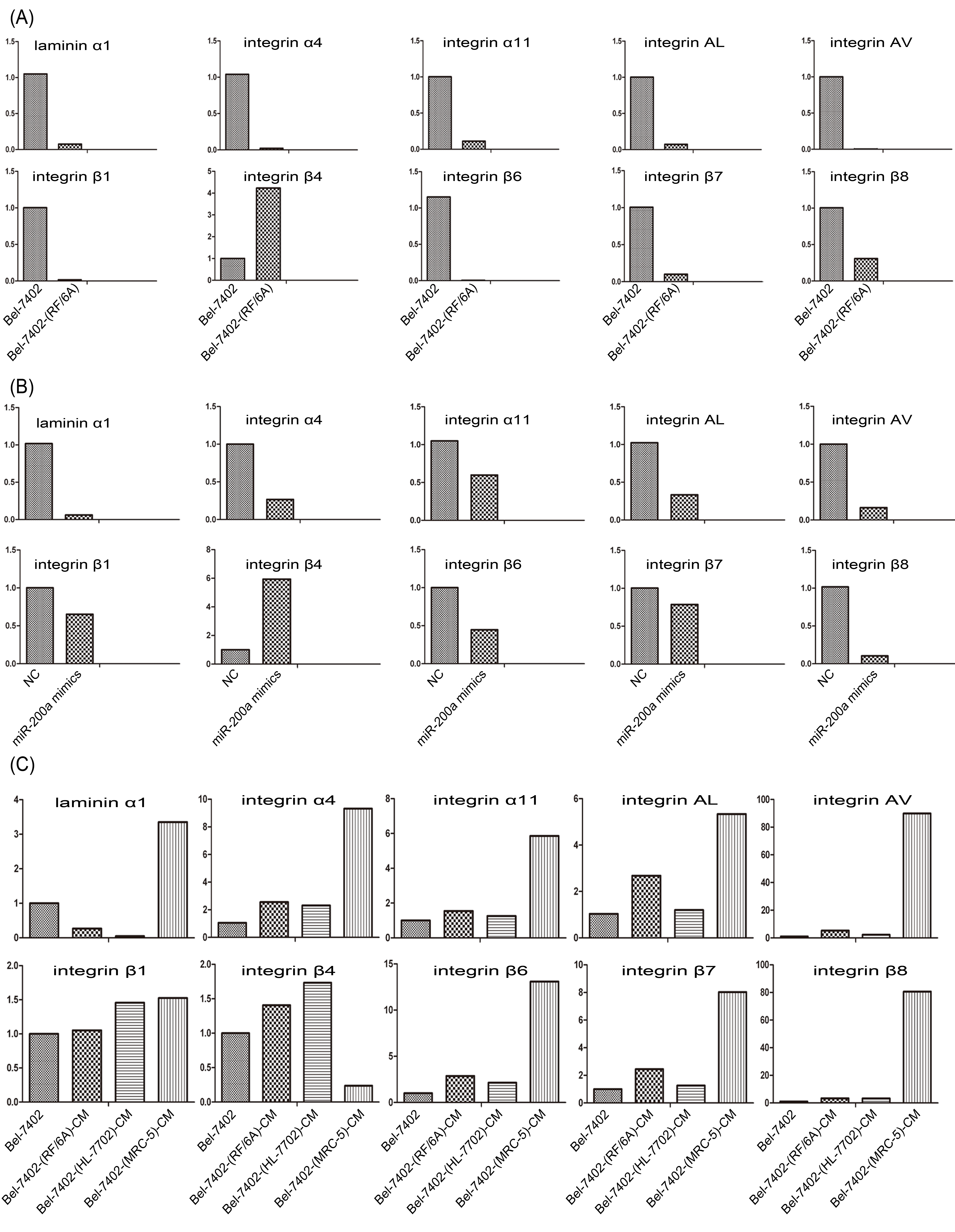

Supplement: Additional file 2: Figure S1 — Laminin and integrin expression. (A) Quantitative RT-PCR results of laminin and integrin in Bel-7402 cells and in Bel-7402 cells co-cultured with RF/6A cells for 90 days. (B) Quantitative RT-PCR results of laminin and integrin in Bel-7402 transfected with negative control or miR-200a mimics. (C) Quantitative RT-PCR results of laminin and integrin in Bel-7402 cells, Bel-7402 cells cultured in conditioned media (CM) of RF/6A cells, Bel-7402 cells cultured in CM of HL-7702 cells, and Bel-7402 cells cultured in CM of MRC-5 cells for 28 days. [file 1479-5876-11-164-S2.tiff]

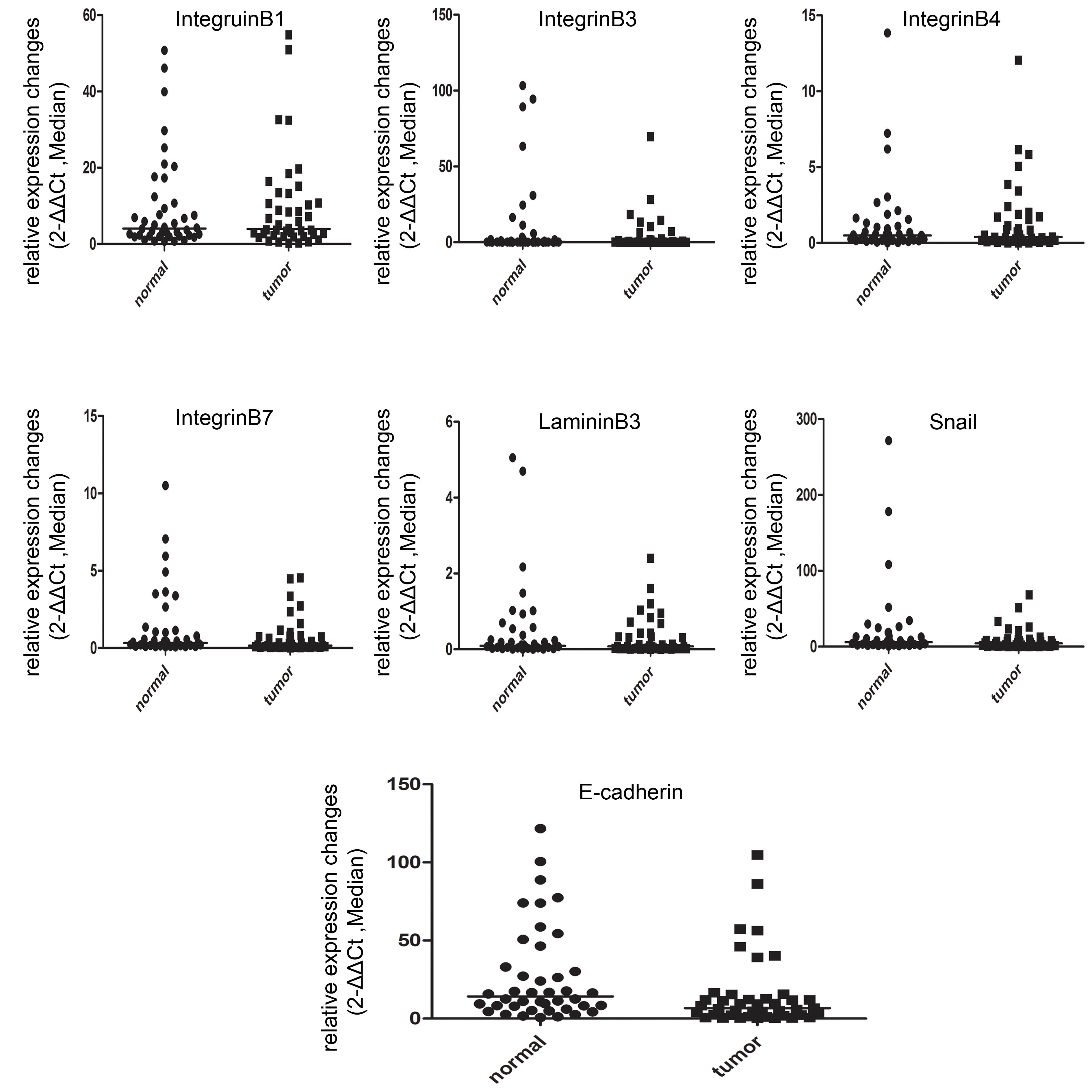

Supplement: Additional file 3: Figure S2 — The relative expression of integrin β1, β3, β4, β7, laminin β3, E-cadherin and Snail in 42 pairs of primary HCC and their adjacent normal tissues. [file 1479-5876-11-164-S3.tiff]

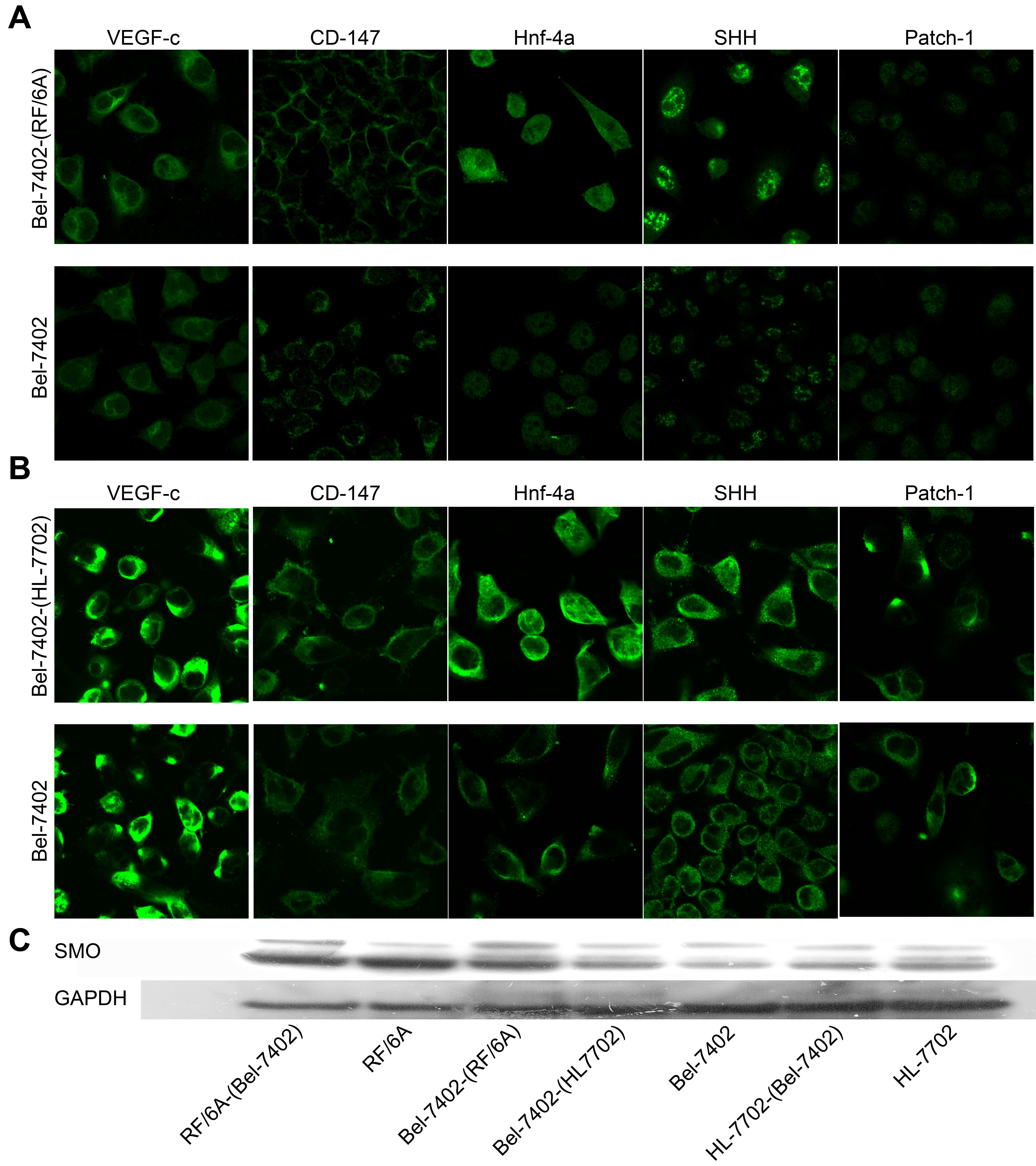

Supplement: Additional file 5: Figure S3 — VEGF-c, CD147, HNF4α, SHH, Patch-1 and SMO expression. (A) Immunofluorescence analysis of VEGF-c, CD147, HNF4α, SHH and Patch-1 in Bel-7402 cells co-cultured with RF/6A cells for 72 days. (B) Immunofluorescence analysis of VEGF-c, CD147, HNF4α, SHH and Patch-1 in Bel-7402 cells co-cultured with HL-7702 cells for 60 days. (C) Evaluation of SMO in co-cultured Bel-7402 cells at day 44. [file 1479-5876-11-164-S5.tiff]
